# Validation of a novel cardiac motion correction algorithm for x-ray computed tomography: From phantom experiments to initial clinical experience

**DOI:** 10.1371/journal.pone.0239511

**Published:** 2020-09-30

**Authors:** Duhgoon Lee, Jiyoung Choi, Hyesun Kim, Minkook Cho, Kyoung-Yong Lee

**Affiliations:** Advanced R&D Team, Health and Medical Equipment Business, Samsung Electronics, Suwon-si, Gyeonggi-do, Korea; University of Oklahoma, UNITED STATES

## Abstract

A novel cardiac motion correction algorithm has been introduced recently. Unlike other segmentation-based approaches it is fully automatic and capable of correcting motion artifacts of myocardial wall and other moving structures as well as coronary arteries of the heart. In addition, it requires raw data of only less than a single rotation for motion estimation and correction, which is a significant advantage from the perspective of x-ray exposure and workflow. The aim of this study is to explore the capability of the proposed method through phantoms and in-vivo experiments. Motion correction of coronary arteries and other heart structures including myocardial wall is the main focus of the evaluation. First, we provide a brief introduction to the concept of the motion correction algorithm. Next we address the procedure of our studies using an XCAT phantom and commercially available physical phantoms. Results of XCAT phantom demonstrate that our solution significantly improves the structural similarity of coronary arteries compared to FBP (proposed: 0.94, FBP: 0.77, *p*<0.001). Besides, it provides significantly lower root mean square error (proposed: 20.27, FBP: 25.33, *p* = 0.01) of the whole heart image. Mocomo phantom study shows that the proposed method improves the visualization of coronary arteries estimated based on motion score (1: worst, 5: best) from two experienced radiologists (proposed: 3.5, FBP: 2.1, *p*<0.001). The results of these phantom studies reveal that the proposed has a great potential in handling motion artifacts of other heart structures as well as coronary arteries. Finally, we provide the results of in-vivo animal and human studies. The 3D and 4D heart images show a consistently superior performance in the visualization of coronary arteries along with myocardial wall and other cardiothoracic structures. Based on these findings of our studies, we are of the opinion that our solution has a considerable potential to improve temporal resolution of cardiac CT imaging. This would open the door to innovations in structural or functional diagnosis of the heart.

## Introduction

Motion artifacts are still one of the major challenges in diagnosis of the heart using computed tomography (CT) despite the latest hardware advancements such as fast gantry rotation speed, wide detector, or dual source. Recently, software approaches [1–6] that could overcome the intrinsic hardware limitations of the CT systems have been introduced. While they showed a good potential by providing promising results, there exist some limitations too. The approaches in [1–3] require 4-D image volumes for motion estimation, which is a heavy computational burden. In addition, they are not directly applicable to the protocols like prospective ECG triggering which acquires single image volume. On the other hand, algorithms of [4,5] are targeting only contrast enhanced coronary arteries and require extra steps of processing such as segmentation, labeling or region of interest (ROI) setting. In the worse cases, some manual jobs are necessary, which is suboptimal from the workflow point of view [4]. Another issue to be pointed out is that some approaches require more data than conventional cardiac short scan, which means an extra x-ray exposure [4] to patients. The approach in [6] makes use of fiducial markers for motion estimation, which is again not optimal from the workflow point of view. Moreover, it is questionable whether we can estimate the motion of the heart by the help of external markers. So far, no approach has been introduced, to the best of our knowledge, to tackle motion correction of both the myocardial wall and coronary arteries using raw data acquired in less than a single rotation.

A novel cardiac motion correction algorithm, sub-cycle universal linear model low-dose imaging (SCULLI), has been introduced recently [7, 8]. Its original concept and capability of motion correction for various heart rates and cardiac phases have been demonstrated in [7–10]. Moreover, studies on its efficacy in calcium scoring and stenosis measurement have been introduced in [11, 12]. The performance of the motion correction in CT scans with 0.33 sec rotation speed was studied lately in [13]. However, no systematic study has been published on the motion correction capability of SCULLI for various heart structures, in which the novelty of this paper lies.

Now we would like to recall the concept of SCULLI briefly. Assuming that a cardiac CT scan has been taken, raw data necessary for image reconstruction at the given target phase is selected in preference. By default, SCULLI needs a gating window centered at the target phase having the width of π+2×fan angle, which is less than a single rotation. After fan-to-parallel rebinning is carried out, two PAR (partial angle reconstruction) images are generated using the first and the last views. These two PAR images are conjugate, namely a half rotation apart. A band pass filtering is applied to the PAR images to extract edge information while discarding artifacts. In case of no motion, these two would match. As is typical in other approaches, a non-rigid registration based on free form deformations [14] is executed to estimate the motion between the filtered PAR images. As a result, we obtain a motion vector field, which enables to acquire motion of each view (or time point) to the target view by a linear assumption. The target is set to the midpoint of the two time points of the PAR images by default. Motion compensated back-projection is performed to acquire result images. We adopted the weighted filtered backprojection (WFBP) in PAR image generation and motion compensated reconstruction. For more details on each step of the processing, please refer to [7, 8].

Although PAR images are not complete, they provide time-resolved edge information of the structures. Furthermore, since there is no segmentation step in the process of building PAR images, myocardial wall, coronary arteries and other structures would leave edge information. This way, we can capture the difference of the edges between two PAR images regardless of the structures and it allows motion estimation between the two time points.

This nature of SCULLI brings up a question: Is SCULLI able to compensate for the motion of other heart structures as well as coronary arteries? This question addresses an important issue since motion artifacts are still unavoidable even in many clinical practices using contemporary cardiac CT systems. Undoubtedly, the ultimate goal of cardiac computed tomography angiography (CCTA) is to acquire images of coronary arteries free from motion artifacts. It was also pointed out that motion artifacts of myocardial wall can mimic ischemic defects in myocardial CT perfusion (CTP) [15]. For this reason, it is also very meaningful to deal with the motion artifacts of myocardial wall.

In this study, we explore the motion correction capability of SCULLI for both coronary arteries and other heart structures, including myocardial wall. We demonstrate the results of phantom experiments, which are followed by those of in-vivo animal and clinical studies.

## Materials and methods

SCULLI consists of three main steps: partial angle reconstruction (PAR), motion estimation, and motion compensated reconstruction [7, 8]. There are several parameters which can influence the performance of the motion estimation and correction. We performed in-house evaluations to select the parameter values and used them for phantom and in-vivo studies. First, the PAR angle was set to 52° corresponding to the fan angle of the prototype CT system. Second, the frequency thresholds of [0.1, 0.3] (unit: π rad/sample) were used for the band pass filter applied to the PAR images. Last, for our motion estimation we have adopted a non-rigid registration technique with 3.6 mm grid space and three multi-resolution steps. Please note that the three main steps and relevant parameters were introduced in [7]. More details regarding the practical meaning of the parameters will be covered in Discussion.

### Various phantom tests

**Fig 1:**
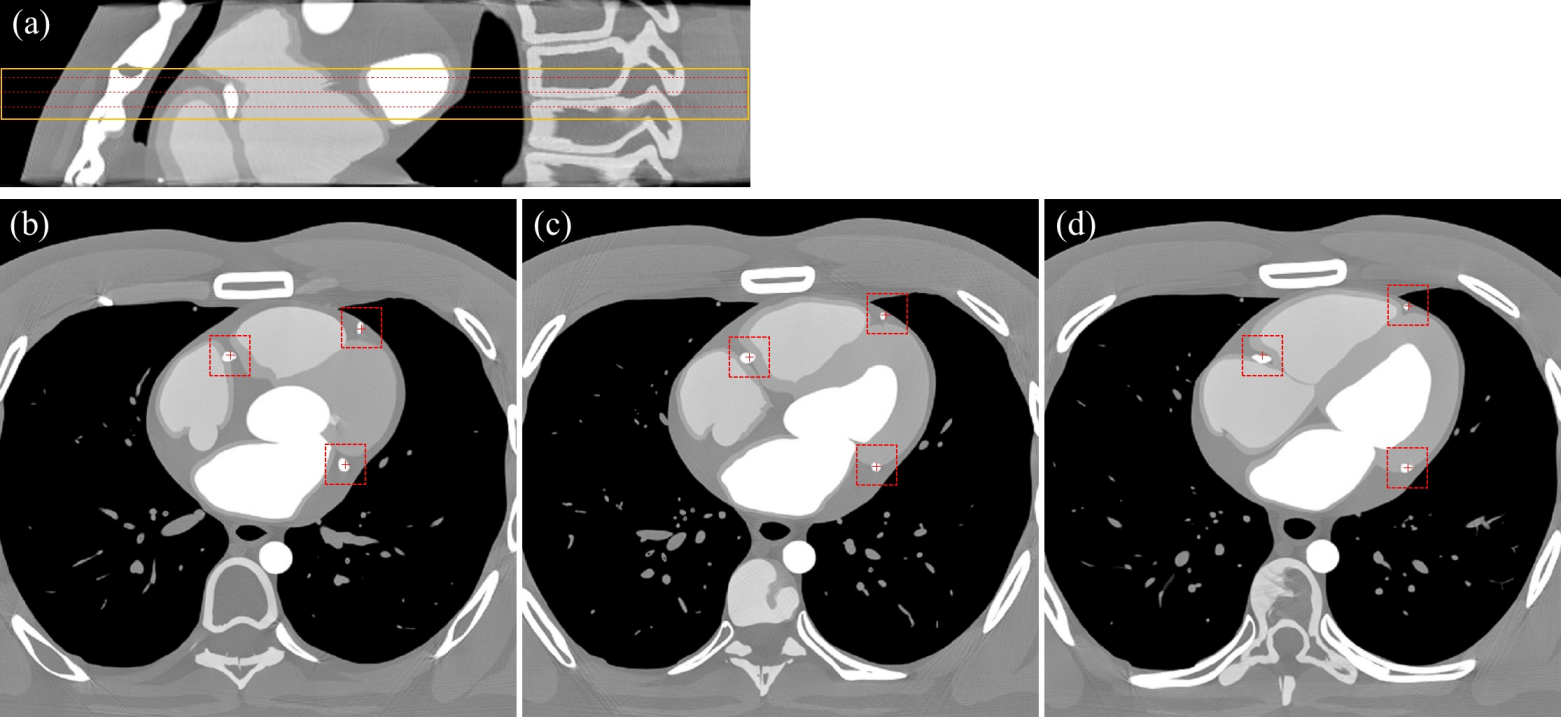
Three slice locations and ROI centers at each coronary artery (static at 30% R-R). (a) Sagittal view of the XCAT phantom. Three red dotted lines and the orange solid line indicate the slice locations and the sub-volume which are used for the calculation of MSSIM and RMSE respectively. (b)-(d) Transaxial images at three slice locations with ROI centers (marked by + sign) and extensions.

In order to evaluate the performance of SCULLI, we conducted various phantom experiments, which can be classified as software phantom test and physical phantom test. First, we utilized the XCAT [16] phantom for the software test. We had a convenient control of the experiment to impose various conditions, such as system geometry, rotation speed, contrast enhancement and the movement of the object. Assuming the geometry of a prototype 128 slice CT system, raw data was simulated for heart rates of 60, 80 and 100 bpm. We also gave a different contrast to the left and right atria and ventricles. No noise was added to explore the pure performance of the algorithm. Images were reconstructed at R-R 30% and R-R 70% phases with 0.625 mm slice thickness and slice interval with 250 mm FOV. Results of FBP and SCULLI were compared quantitatively using the ground truth. First, we selected three small ROI regions at each slice location (three slice locations in total) so that each ROI includes right coronary artery (RCA), left anterior descending (LAD), and left circumflex artery (LCX) respectively. The mean structural similarity (MSSIM) [17] was calculated at each ROI to evaluate the severity of motion artifacts of the coronary arteries as described in Fig 1.

On the other hand, root mean square error (RMSE) was calculated using the whole images of the sub-volume described in Fig 1. (a) to quantify the motion artifacts of the whole heart structure. Moreover, the stability of Hounsfield Unit (HU) was measured along a profile on a uniform region of the myocardial wall. A single sided paired t-test was performed between MSSIM values, and RMSE values from FBP and SCULLI images respectively. A value of *p*<0.05 was considered significant.

**Fig 2:**
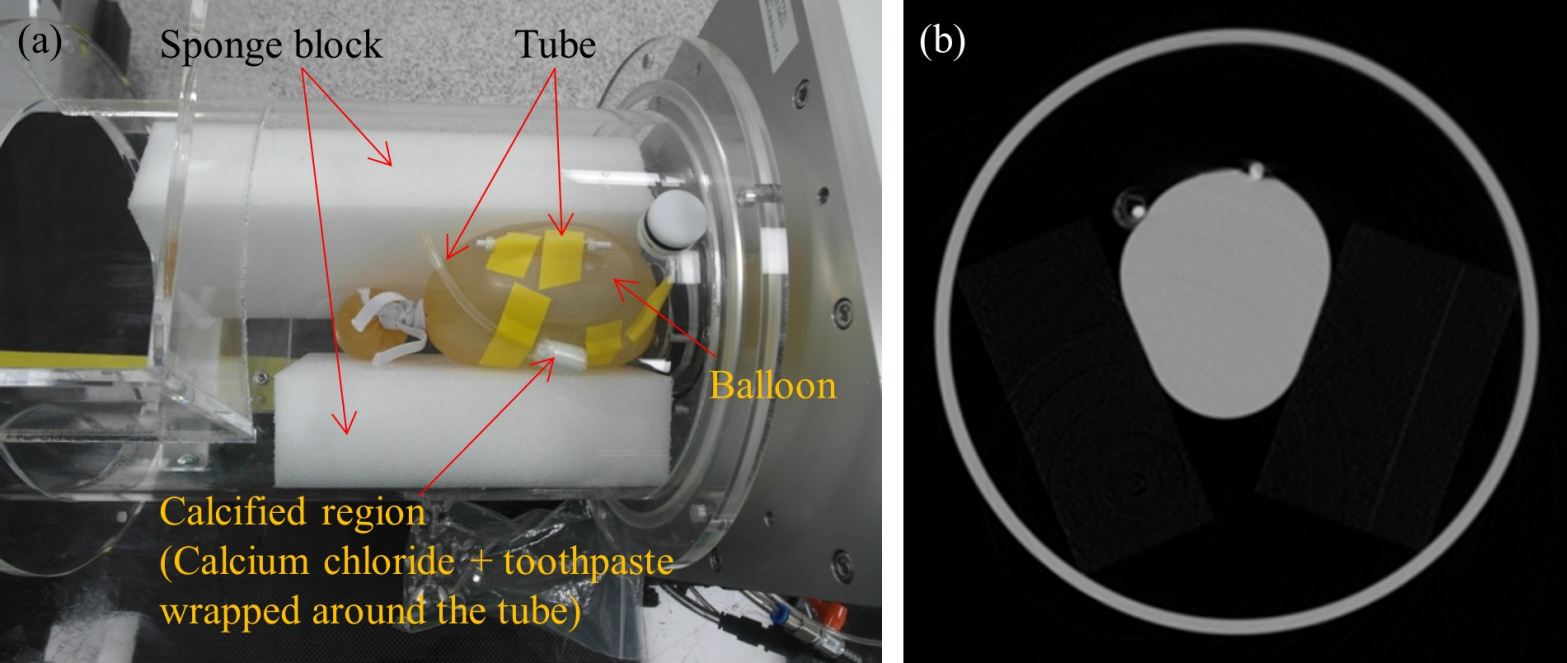
CT scan setup of the Alpha phantom. (a) In order to mimic heart chamber (or myocardial wall), coronary artery and calcified artery, and several structures were placed. (b) The structures without motion are clearly observed in the CT image.

**Fig 3:**
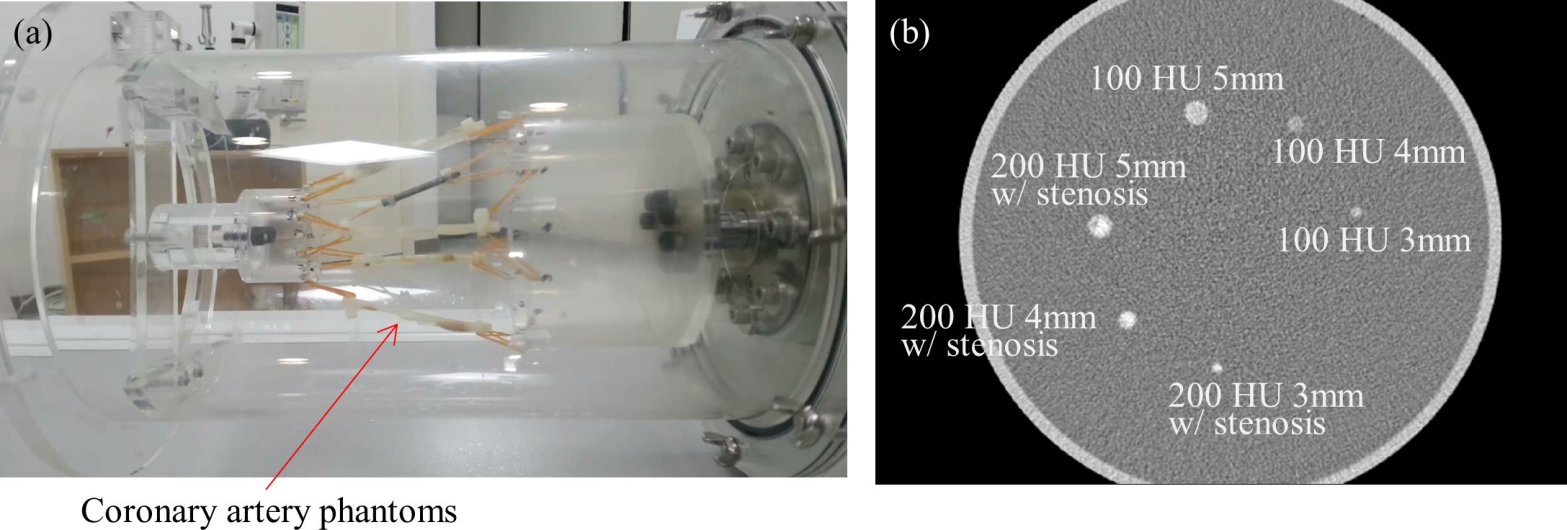
CT scan setup of the Mocomo phantom. (a) Various coronary artery phantoms with different sizes, intensities, and shapes were installed. (b) The static arteries are clearly visible in CT image.

XCAT has clear limitations, such as ideal system geometry, simple anatomical structures and movement, despite its convenience. For these reasons, we designed moving phantom experiments at CT systems as the next stage. The goal was to test SCULLI under more realistic and challenging conditions. We customized two commercially available phantoms, namely Alpha (Alpha1-VTPC, Fuyo) and Mocomo (Mocomo1, Fuyo). The pulsating phantom Alpha is designed to mimic heart chamber (or myocardial wall) and coronary artery motion (see Fig 2) while Mocomo simulates coronary artery motion by a rotational and translational movement (see Fig 3). Both phantoms generate ECG signals to be displayed in ECG monitors and stored in the computer connected to the phantom. The ECG signal and the raw data were synchronized retrospectively.

First, we placed two sponge blocks under the water-filled balloon at both sides in Alpha phantom. They are designed to support the balloon and make the motion non-uniform along the radial direction of the balloon. Two artery phantoms were attached on the surface of the balloon and designed to move as the balloon contracts and expands repeatedly. We diluted iodine with water so that the water in the balloon has a clear contrast of 250~300 Hounsfield Unit (HU). Axial scans were taken on BodyTom (Neurologica, USA) under 120 kVp and 150 mA for the heart rates of 20, 25, 30 and 35 bpms. Images were reconstructed under 1.25 mm slice thickness and slice interval with 250 mm FOV at various R-R phases. The visibility and motion artifacts of the whole phantom structure, namely heart chamber (or myocardial wall) and coronary arteries, were evaluated by a visual inspection of experienced engineers.

**Fig 4:**
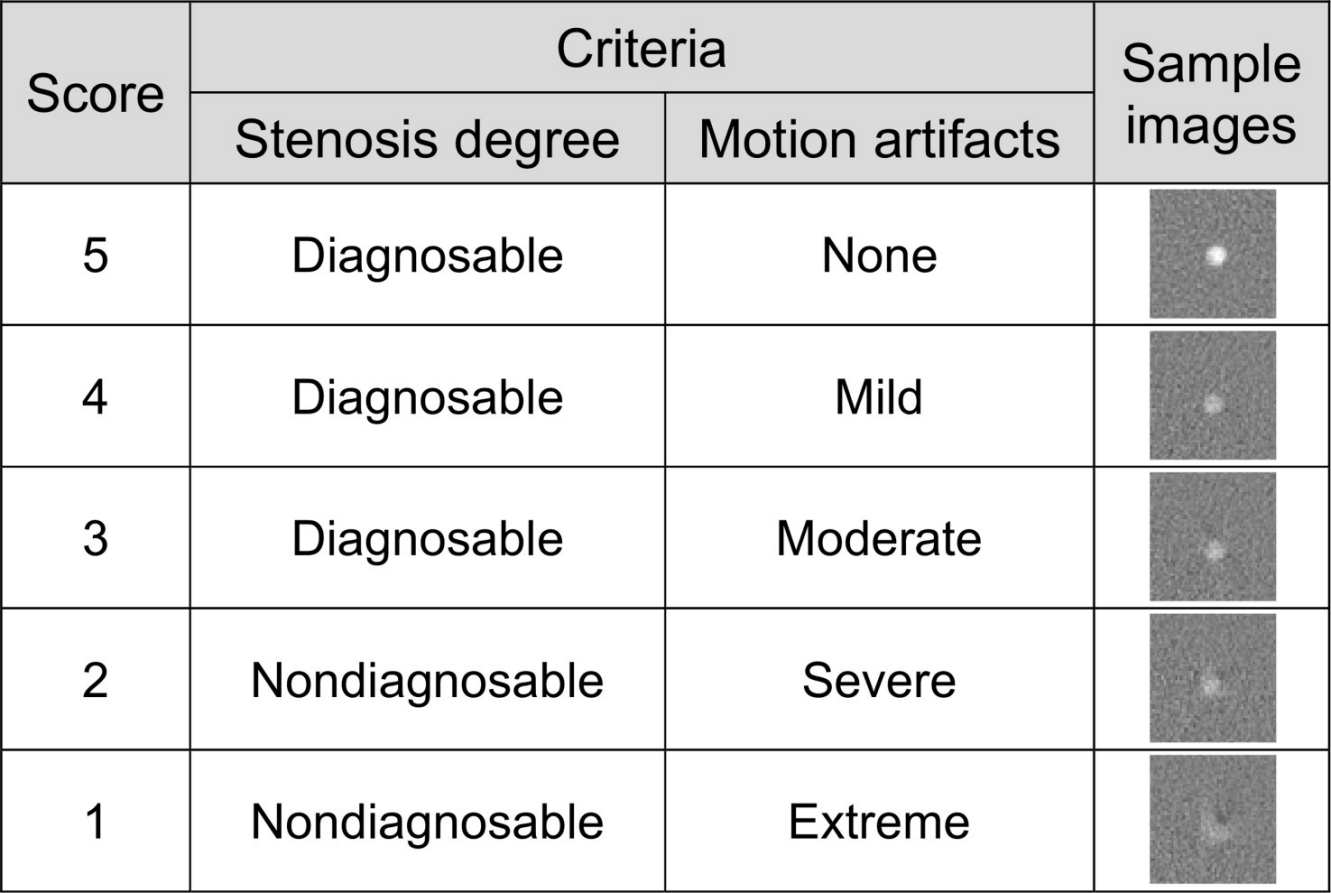
Criteria of the motion score.

Next, coronary arteries phantoms with and without stenosis were installed in the Mocomo phantom as depicted in Fig 3. Total six artery phantoms with diameter 3, 4 and 5 mm were scanned on BodyTom with the heart rates of 15, 25 and 35 bpms. We had to conduct the experiment with lower bpms, as the system’s default rotation speed was one second per rotation. Assuming a CT system with 0.25 sec/rotation, the heart rates would correspond to 60, 100 and 140 bpms. Scans were taken under 120 kVp and two different currents, namely 50 mA and 200 mA. These conditions correspond to 11.83 mGy and 47.32 mGy respectively. Images were reconstructed by FBP and SCULLI at R-R 70% phase with 1.25 mm slice thickness and slice interval with 250 mm FOV. Two experienced radiologists (cardiac experts with 12 and 17 years experiences respectively) evaluated the performance according to the so-called motion score (1: worst, 5: best), namely five point Likert scale. The score was defined based on two criteria. One is the severity of the motion artifacts and the other is being able to make a diagnosis of stenosis degrees as given in Fig 4.

A paired t-test was again adopted to evaluate the difference between the scores of FBP and SCULLI. A value of *p*<0.05 was considered significant. The average scores of the two radiologists were utilized for the evaluation of both FBP and SCULLI. To assess intraobserver agreement Cohen’s kappa was calculated [18].

Although physically moving phantoms provide a good opportunity to test SCULLI by using real raw data, they have some limitations at the same time. First, the overall movement of the coronary arteries is repetitive and patterned due to the mechanical design of the moving phantoms, which is not the case for the real heart. Due to this limitation we considered various heart rates and heart phases instead of repeating the experiment under the same condition. As it is well known, the three coronary arteries, namely RCA, LAD, and LCX, are different in the amount of the motion and velocity due to the complex and dynamically squeezing movement of atria and ventricles [19–23]. Second, injection of a contrast agent and the flow of contrast enhanced blood were not able to be considered in the physical phantoms, which can potentially affect the motion estimation due to the dynamic change of HU values. These are the reasons why we need in-vivo studies which are addressed in the next section.

### In-vivo animal and clinical studies

An in-vivo animal study was designed to explore the potential of SCULLI. This study was reviewed and approved (Approval number: 20140911003) by the Institutional Animal Care and Use Committee (IACUC) of Research Institute for Future Medicine (RIFM) at Samsung Medical Center (SMC). A swine underwent a CT scan according to a prospective ECG triggering protocol of 128 slice prototype CT system with a padding option. Detailed scan conditions are given in the Table 1.

**Table 1:** Scan protocols of the in-vivo animal and clinical studies.

	Protocol	kVp	mA	Rotation speed (sec/rotation)	Collimation (mm)	Padding (R-R %)	CTDIv (mGy)
Swine	Prospective ECG triggering	100	300	0.25	80	31~81	8.07
Case A		100	700	0.25	80	30~80	9.28
Case B		100	700	0.25	80	60~80	9.28
Case C (Calcium Scoring)		120	400	0.25	80	40	5.99

Reconstruction was carried out with 0.625 mm slice thickness and interval, 250 mm FOV. The mean heart rate was 85 bpm. Images were generated by FBP and SCULLI at various R-R phases and then evaluated by a visual inspection. In addition, the stability of HU values was assessed with the values measured on a line profile throughout five different cardiac phases.

Finally, we conducted an initial clinical study which has been IRB approved (Approval number: SMC 2015-06-066-001) in Samsung Medical Center (SMC). Scan protocols were prospective ECG triggering with or without padding option under 0.25 sec/rotation and 128 slice collimation. For detailed scan protocols, please refer to Table 1. We chose the three cases retrospectively. The average heart rates were 63 bpm (Case A), 54 bpm (Case B) and 95 bpm (Case C). Images of Case A and B were reconstructed by FBP and SCULLI at various R-R phases using the same slice thickness and interval. Images were reformatted in 3D image volumes and curved multiplanar reformation (MPR) for evaluation of the coronary arteries and other heart structures. In addition, 4D cine images were generated to evaluate the functional performance. As for Case C, FBP and SCULLI were carried out at 40% with 2.5 mm slice thickness and interval. The presence of motion artifacts of calcified regions was investigated.

## Results

### Results of phantoms experiments

**Fig 5:**
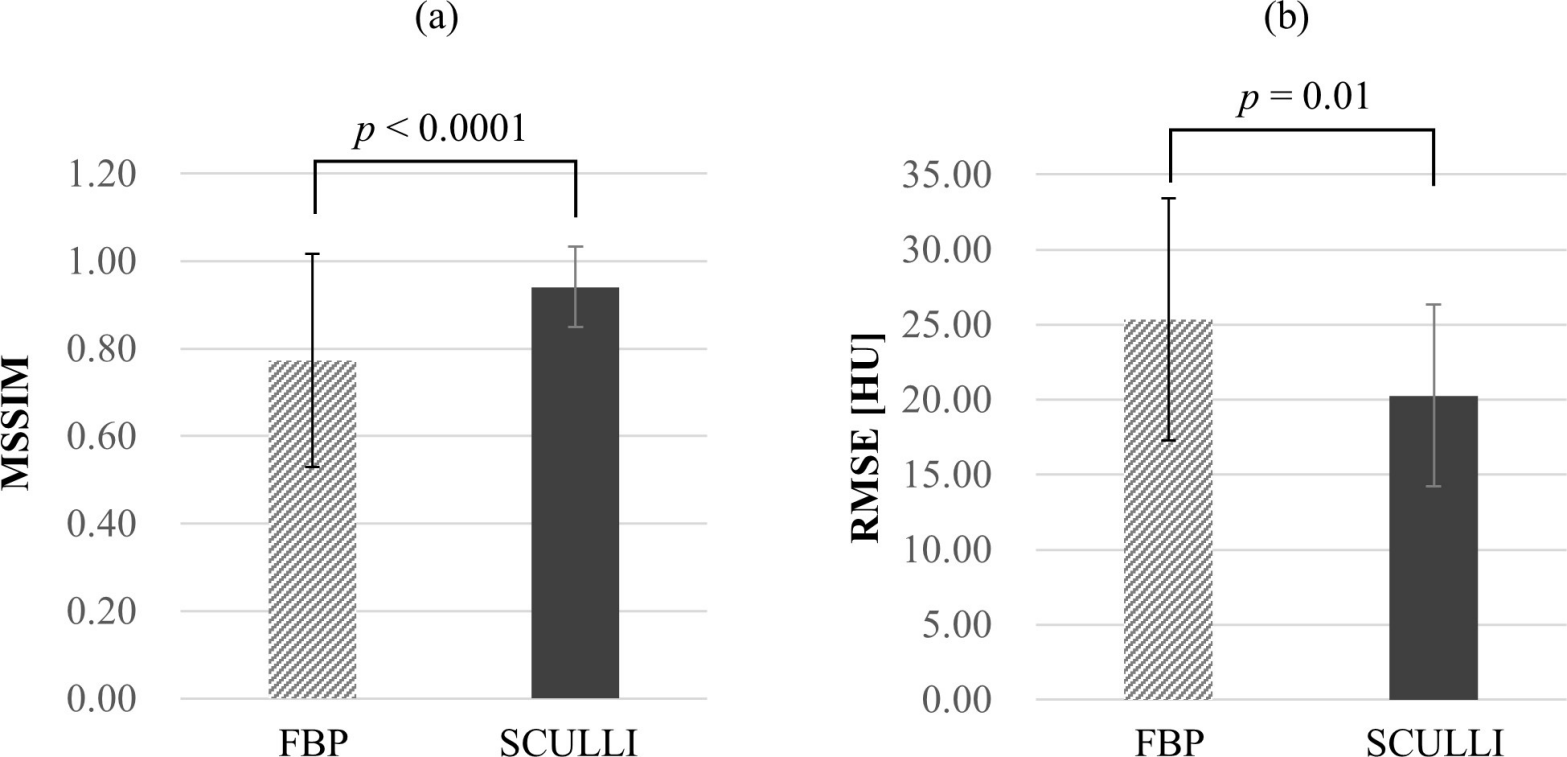
Quantitative evaluation of SCULLI for XCAT phantom. Quantitative analysis shows a significant improvement (paired t-test) of the average (a) MSSIM and (b) RSME across all the ROIs, heart rates (60, 80, 100 bpm), and cardiac phases (R-R 30% and 70%) of SCULLI images compared to FBP images.

For the heart rates of 60, 80, and 100 bpms, we assessed the performance of SCULLI at systolic and diastolic phases with the help of XCAT simulation. The overall performance is summarized in Fig 5.

**Fig 6:**
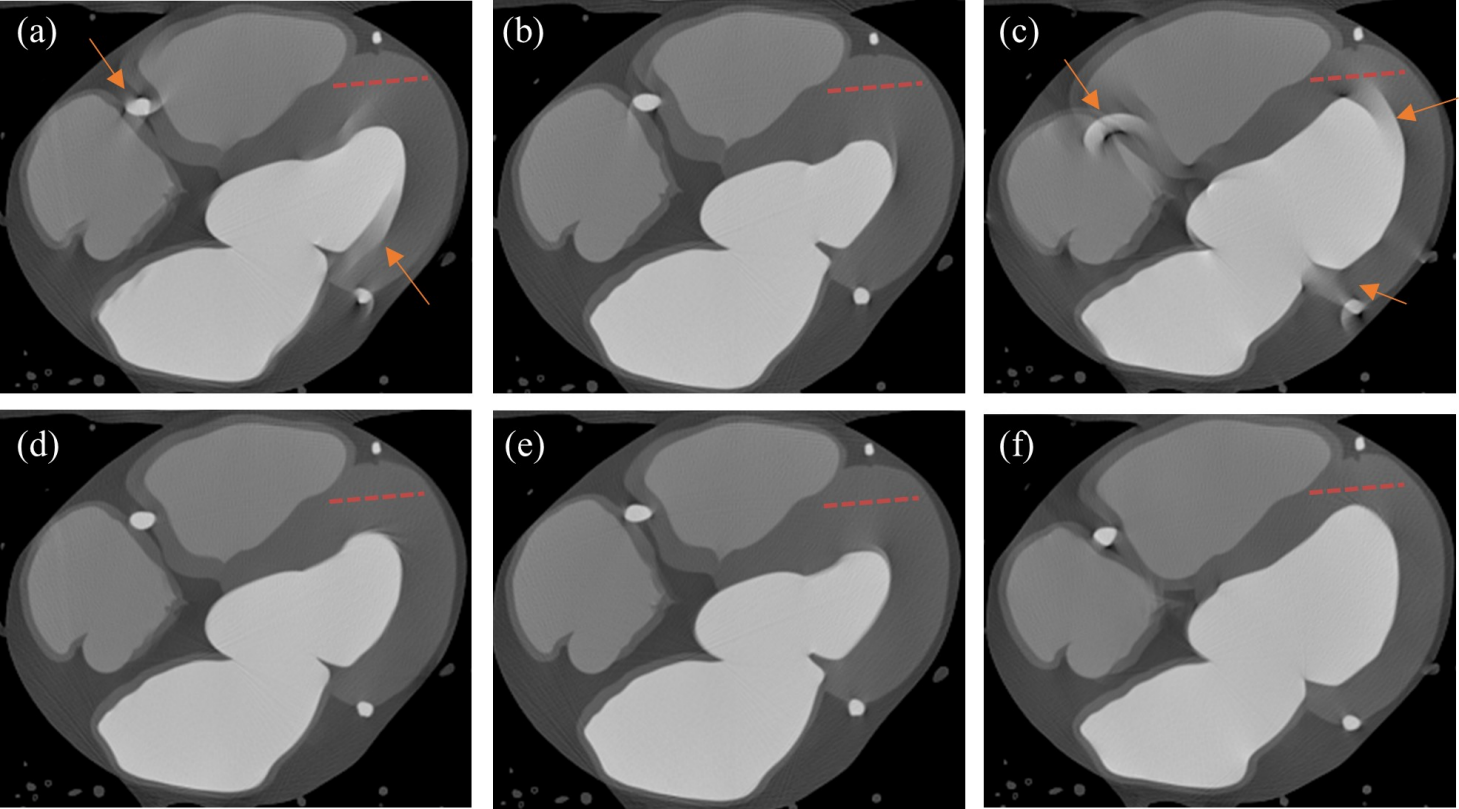
Results of SCULLI for 80 bpm XCAT phantom. Top: FBP at (a) 30%, (b) 50%, and (c) 70% of R-R phase. Bottom: (d)-(e) The corresponding results of SCULLI. (L/W 100/300) The orange arrows in (a), (c) indicate the severe motion artifacts of coronary arteries, ventricles, and myocardial wall in FBP. The motion contaminated structures have been restored by SCULLI nicely as shown in (d), (f).

**Fig 7:**
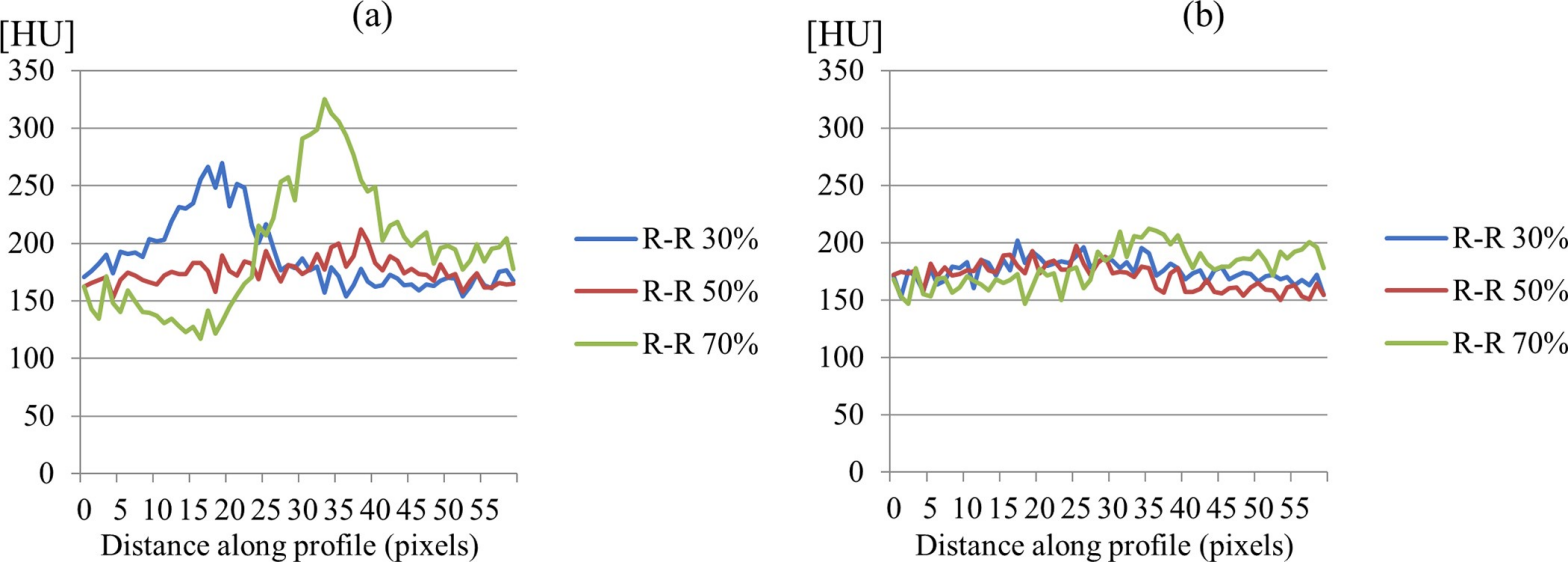
Intensity profiles in myocardial wall of XCAT results. (a) Intensity profiles of FBP on the dashed lines in Fig 6. (b) Corresponding profiles of SCULLI. The results of SCULLI show remarkably more stable HU values compared to those of FBP.

It is observed that the average MSSIM value of SCULLI is clearly higher than that of FBP, namely 22% improvement (SCULLI: 0.94, FBP: 0.77, *p*<0.001). The same tendency is also apparent in RMSE values of the whole heart structure. SCULLI showed 20% less error than FBP on average (SCULLI: 20.27, FBP: 25.33, *p* = 0.01). In Fig 6, we can see time-resolved coronary arteries, myocardial wall, atria and ventricles. Line profiles on a uniform region in myocardial wall reveal in Fig 7 how SCULLI delivers stable HU values throughout R-R cycles compared to FBP.

SCULLI maintains the stability of HU values in the range of [150,200] even at the systolic and diastolic phases unlike FBP whose HU values change drastically in [100, 300], which is around four-fold wider. The standard deviation of all values drops from 29.5 (FBP) to 13.9 (SCULLI).

**Fig 8:**
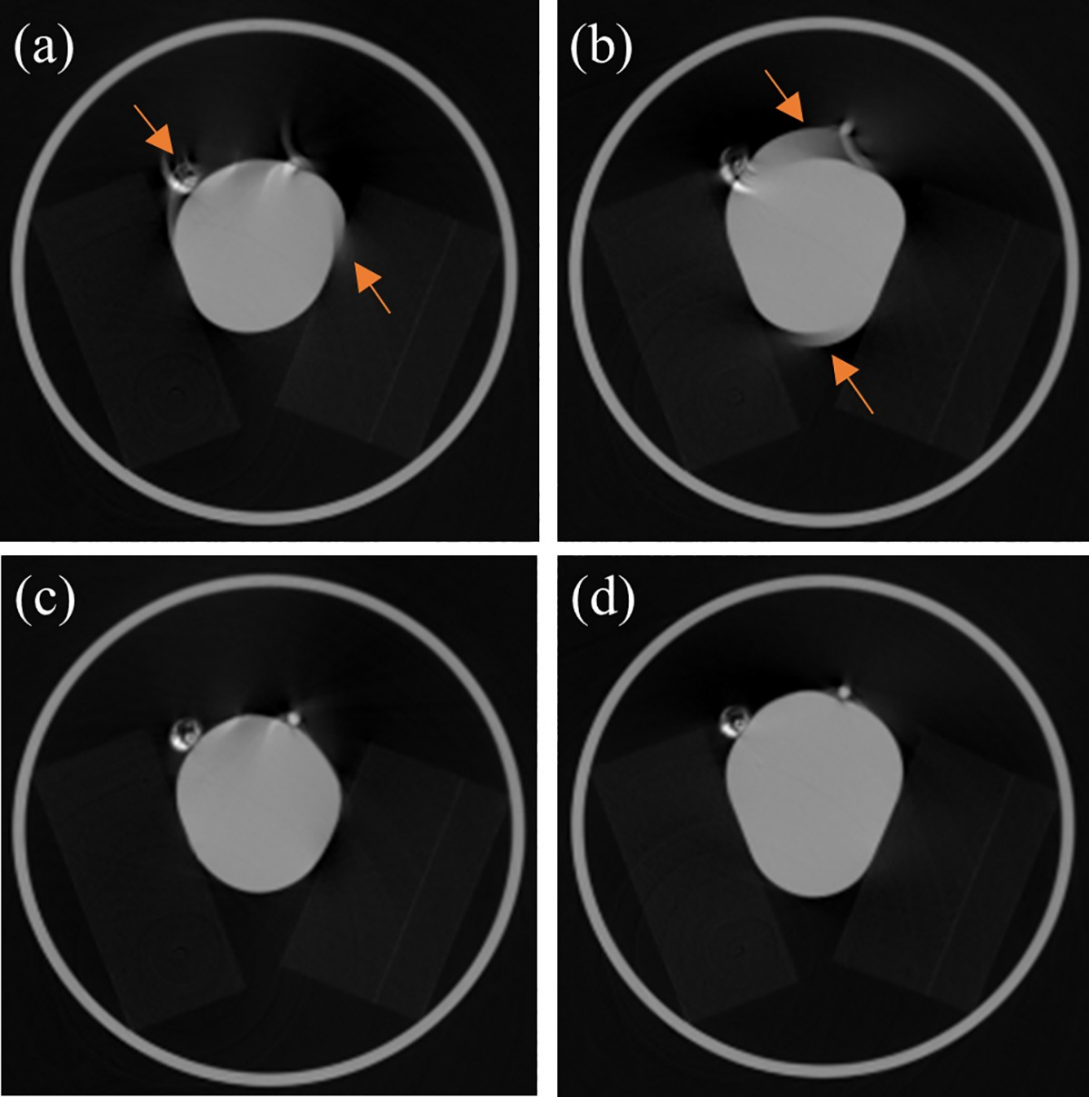
Results of experiment using Alpha phantom (25 bpm). Top: FBP at (a) a systolic and (b) a diastolic phases. Bottom: (c)-(d) Corresponding results of SCULLI (L/W 0.2/0.4 cm^-1^). Motion artifacts of coronary artery phantoms and the balloon (mimicking heart chamber or myocardial wall) indicated by the orange arrows in (a) and (b) are remarkably reduced by SCULLI.

The result of the experiment using Alpha phantom demonstrates that motion artifacts of the coronary arteries and the heart chamber (or myocardial wall) are reduced significantly by SCULLI. In other words, typical motion artifacts, such as the so-called tails of coronary arteries, doubling edges and streaks of heart chamber (or myocardial wall) are significantly reduced as displayed in Fig 8.

**Fig 9:**
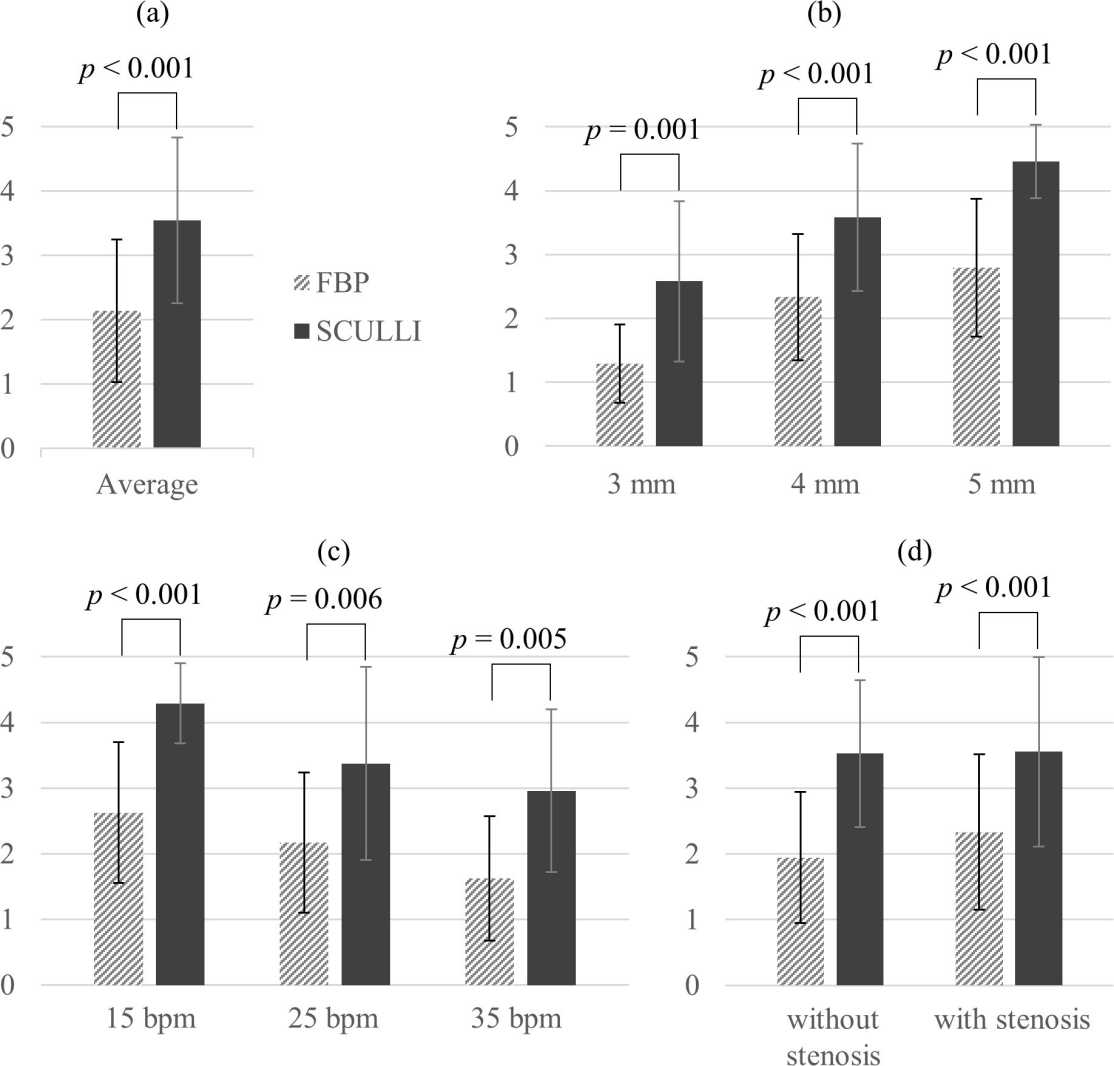
Motion scores of coronary arteries from Mocomo phantom experiment. The average motion scores of two radiologists for (a) all cases, (b) different sizes of diameter, (c) different bpms, and (d) different types (stenosis). In all cases, SCULLI shows a significant improvement.

When it comes to the results of Mocomo phantom study, we have a dramatic improvement of the motion score of SCULLI compared to FBP as in Fig 9.

There was a statistically significant improvement of the average score by the help of SCULLI, namely from 2.1 to 3.5 (*p*<0.001). In other words, SCULLI tends to make the non-diagnosable arteries diagnosable. The score for 3 mm artery is from 1.3 to 2.6 (*p* = 0.001), 4 mm artery is from 2.3 to 3.6 (*p*<0.001), and 5 mm artery is 2.8 to 4.5 (*p*<0.001). The same tendency is observed consistently throughout the heart rates from 15 bpm to 35 bpm. The scores of arteries with and without stenosis were also significantly improved by SCULLI. Note that the SCULLI score of 3 mm artery is less than 3, which is not clinically acceptable despite the significant improvement. The calculated *κ* value was 0.49, showing a moderate agreement of the two radiologists.

**Fig 10:**
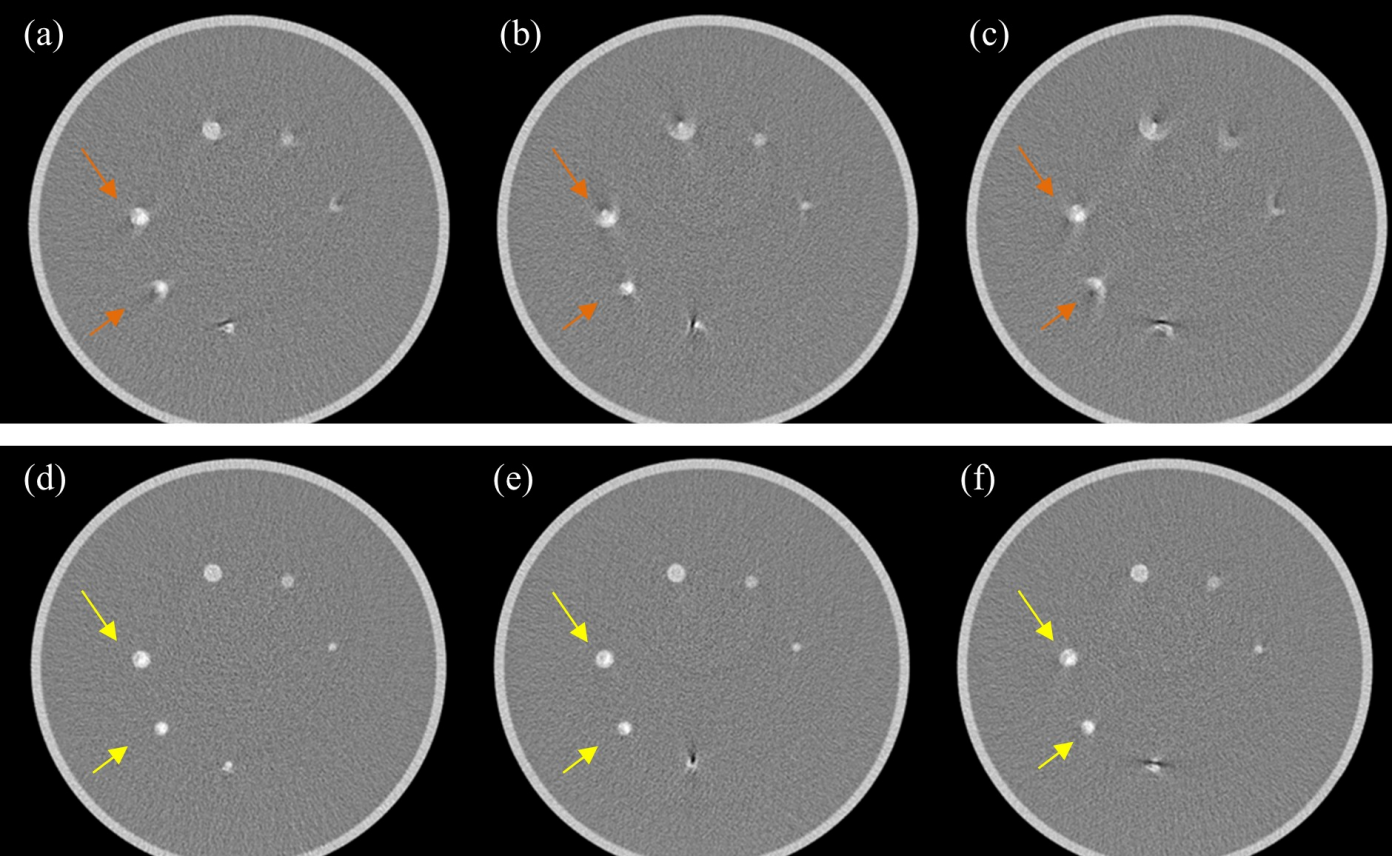
Result of Mocomo phantom experiment. Top: FBP images of moving arteries at (a) 15 bpm, (b) 25 bpm, and (c) 35 bpm show severe motion artifacts (L/W 0/400). Their equivalent heart rates to 0.25 second gantry rotation are 60 bpm, 100 bpm, and 140 bpm, respectively. Bottom: The corresponding results of SCULLI are shown in (d), (e), and (f). The 4 mm, 5 mm arteries of stenosis are clearly visible in the images of SCULLI (yellow arrows) which is not the case in those of FBP (orange arrows).

The transaxial images in Fig 10 show the remarkable performance of SCULLI for six artery phantoms of various diameters and bpms. Note that the arteries with stenosis, in particular 5 mm (9 o’clock) and 4 mm (7 o’clock), are clearly visualized by SCULLI unlike FBP.

### Results of in-vivo animal and clinical studies

**Fig 11:**
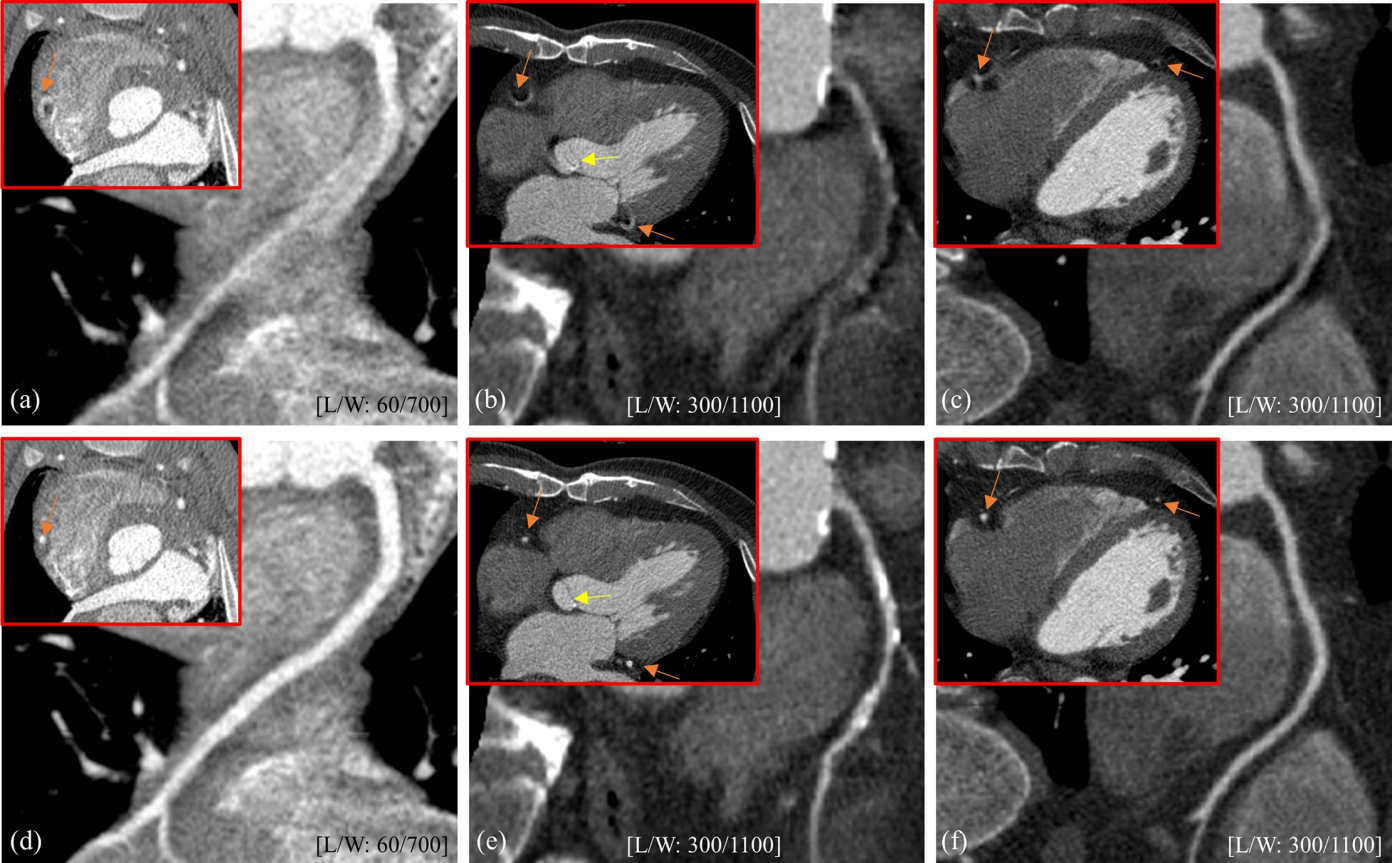
Results of in-vivo cases. Top: FBP images of (a) swine (85 bpm, 70%), (b) case A (63 bpm, 33%), and (c) case B (54 bpm, 61%). Bottom: Corresponding results of SCULLI. RCA in curved MPR shows significantly reduced motion artifacts by SCULLI in (d)–(f). In addition, the aortic valve (yellow arrow) in (e) shows a remarkable improvement by SCULLI.

The result of an animal study shows, even under the higher heart rate of 85 bpm, a promising performance of SCULLI in visualization of RCA in Fig 11(A) and 11(D).

The other clinical cases show a significant improvement of the temporal resolution after applying SCULLI as well. In Fig 11(B) and 11(E) not only RCA, LCX but also aortic valve in the result of SCULLI demonstrate a remarkable improvement in visualization compared to that of FBP. Furthermore, the severe calcification of RCA is nicely visualized in Fig 11(E), which is not the case in Fig 11(B). The result of case B also shows that the motion artifacts of RCA and LAD are considerably corrected in Fig 11(C) and 11(F).

**Fig 12:**
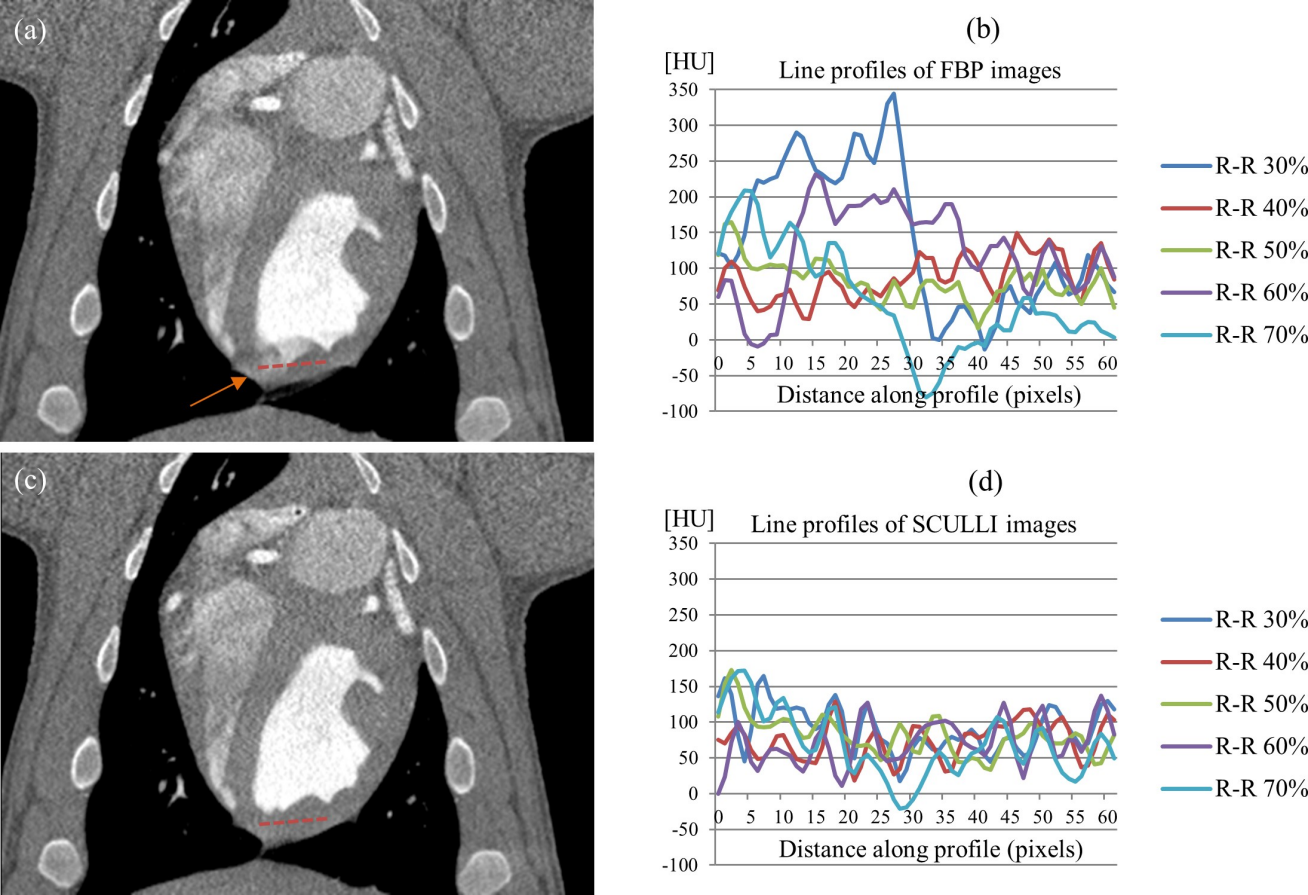
Myocardial wall images of a swine (85 bpm). Coronal images of (a) FBP and (c) SCULLI at 30% of R-R phases (L/W 60/700). HU bias at myocardial wall near the left ventricle (LV) in (a) is observed as a result of the motion of the heart (orange arrow) which was remarkably reduced by SCULLI in (c). Line profiles along the dashed lines at various R-R phases are provided in (b) and (d). The standard deviation of the whole values corresponds to 72.2 (FBP) and 34.1 (SCULLI).

When it comes to myocardial wall motion, Fig 12 shows significantly reduced HU bias caused by heart motion of the swine. The line profile in Fig 12(D) shows that SCULLI delivers much more stable HU values than the conventional FBP throughout the R-R phases. SCULLI shows 53% less standard deviation than FBP.

**Fig 13:**
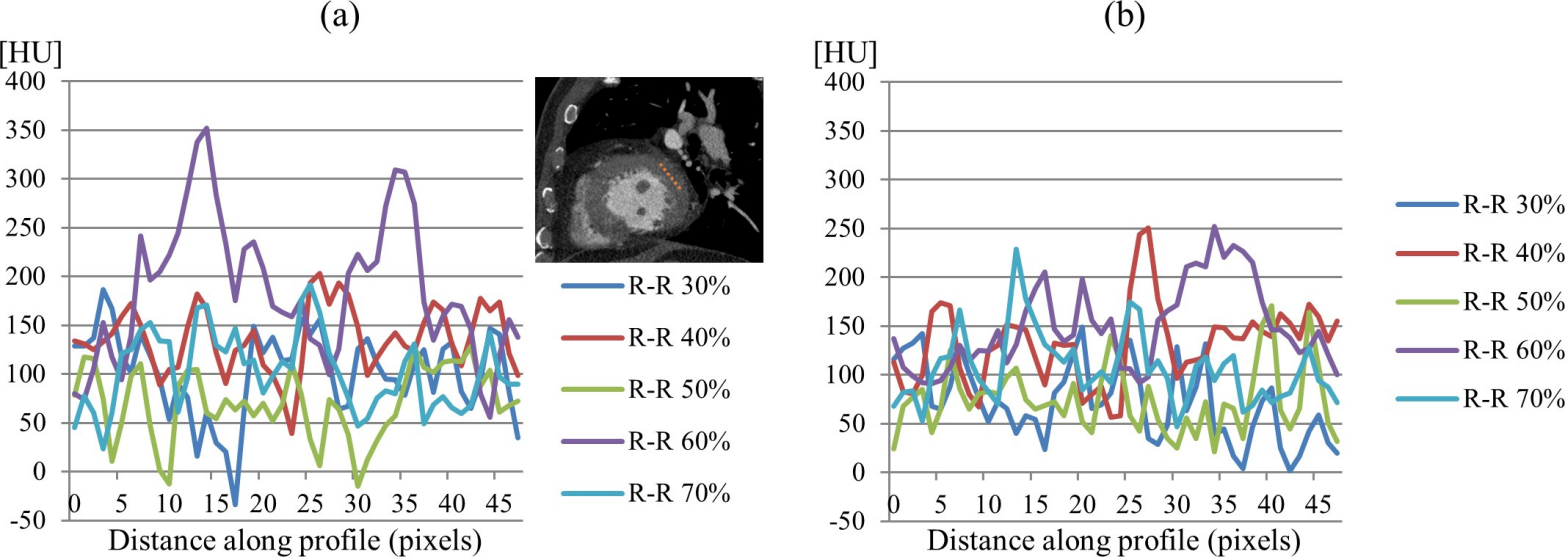
Intensity profiles in myocardial wall of case B. Intensity profiles on a dash line in myocardial wall are plotted for different phases of (a) FBP images and (b) SCULLI results. Standard deviation of SCULLI is 49.5 whereas that of FBP is 60.9.

Moreover, the same tendency is observed in clinical case B as described in Fig 13.

Cine images of the aortic valve of case B in S1 and S2 Videos show the outstanding performance of SCULLIL, which shows closing and opening movements with much higher temporal resolution. Similarly, cine images of the myocardial wall show drastically reduced shadings by SCULLI in S3 and S4 Videos.

**Fig 14:**
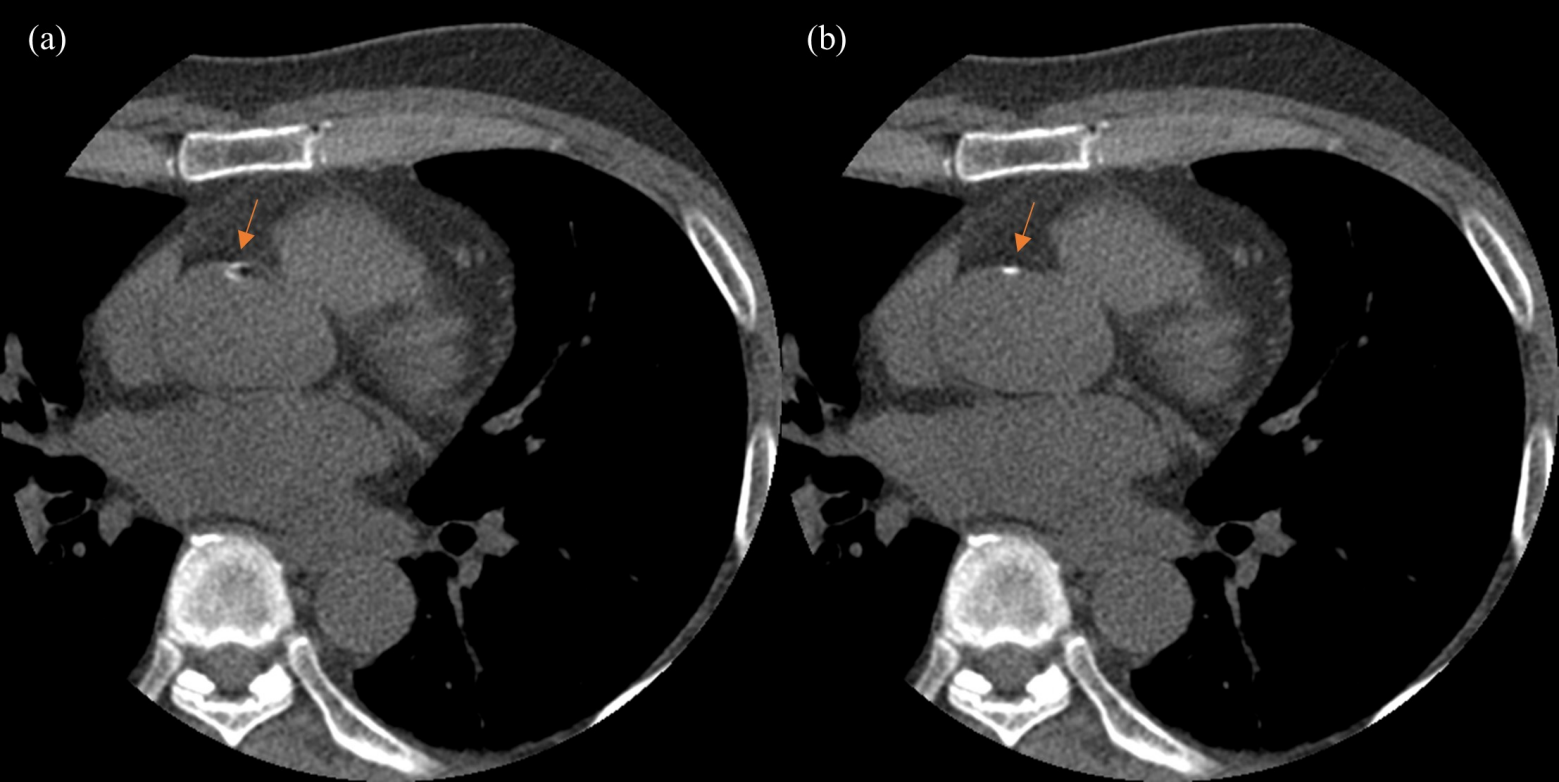
Performance of SCULLI in non-contrast cardiac CT scan (95 bpm, Case C). (a) FBP at 40% R-R shows a calcified region in the ascending aorta with motion artifacts. (b) SCULLI shows the region without motion artifact despite the higher heart rate of 95 bpm (L/W 100/700).

In the result (see Fig 14) of case C (calcium scoring), we were able to see a small calcified region in the ascending aorta with apparent motion artifacts, which have been corrected considerably by SCULLI.

**Fig 15:**
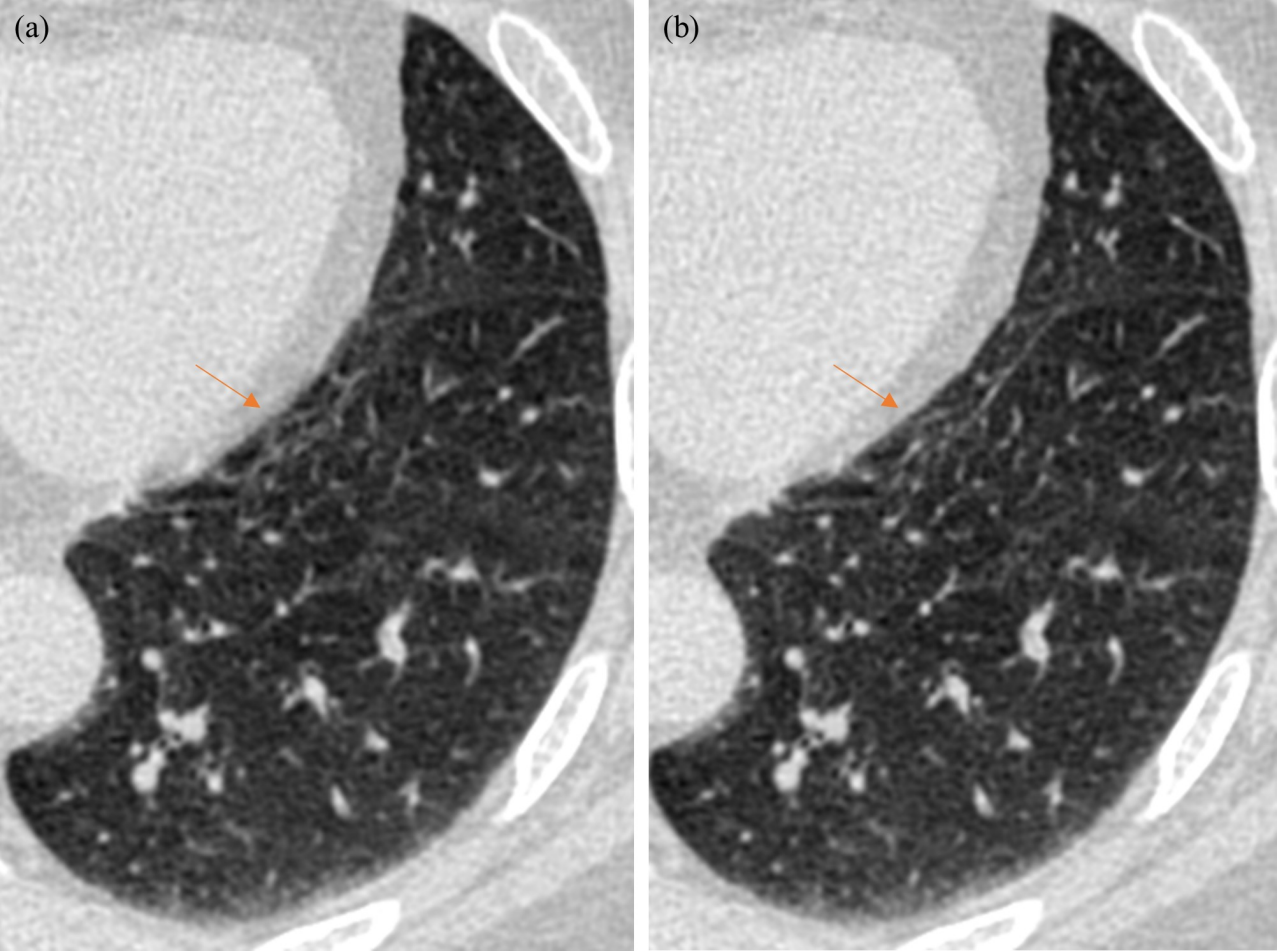
Motion correction performance of SCULLI in lung structures (95 bpm, Case C–Calcium scoring). (a) FBP at 40% of R-R shows motion artifacts of lung near LV. Membrane and the lung structures are degraded by the motion of the neighboring heart. (b) SCULLI shows the structures clearly. (L/W -400/1200).

Investigation of the lung structure in lung window setting reveals the capability of the SCULLI as well. As presented in Fig 15, the distorted membrane of the lung near LV (Case C–Calcium Scoring) is recovered by SCULLI together with neighboring lung structures.

## Discussion

In this paper, we presented the capability of SCULLI by means of phantom and in-vivo studies. SCULLI showed a consistent performance in correcting motion of coronary arteries and other heart structures including myocardial wall. Furthermore, SCULLI has several advantages over the aforementioned software approaches. Namely, it requires raw data from less than a rotation and it is free of segmentation. These benefits enable SCULLI to open the door to new possibilities in cardiac CT. At the same time there are some limitations to be addressed.

### Limitations of the study

The parameter values utilized for this study were fixed as a result of in-house optimization procedure, which is beyond the scope of this paper. However, it would be worthwhile dealing with some background. First, we start with the PAR angle. There is a tradeoff between the completeness of edge information and the temporal resolution of the PAR images. In other words, a larger angle for PAR is advantageous due to rich structural information, but at the same time, it is prone to generate motion contaminated PAR images, which makes motion estimation challenging. A smaller angle, on the other hand, can generate PAR images free of motion artifacts, but the motion of missing edges could be inaccurate. Therefore, it is important to determine an appropriate size of the partial angle for the sake of accurate motion estimation. Currently, we have fixed the PAR angle as the fan angle of the CT system. Further evaluations for various conditions, such as heart rates, rotation speeds of the CT system, will determine the sweet spot of the PAR angle. Second, we have a band pass filtering after PAR image generation. This was adopted to suppress the artifacts [7, 8] appearing as shading artifacts around high contrast objects in PAR images. It seems that the frequency thresholds [0.1, 0.3] deliver the best results so far. Again, it needs to be explored whether this is valid for various target structures, such as coronary arteries, valves, and myocardial wall. Third, the non-rigid registration adopted for our motion estimation is based on a B-spline free form deformation that has a multi-resolution approach [14]. The grid space between two control points and the number of multi-resolution steps in the non-rigid registration can affect the maximum amount of motion to be captured between two images. This is again linked to the maximum displacement of structures in the heart at two different time points at which PAR images are generated. Many studies have been conducted to explore the nature of the movement of coronary arteries [19–23]. Based on the literatures, we defined the maximum displacement of coronary artery between two PAR images as 14.3 mm per 125 msec of half gantry rotation. This seems quite reasonable taking into account a similar specification in [5]. The parameter set with 3.6 mm of grid space and 3 of multi resolution steps showed the best performance, however, we need to explore further to find the optimal values for various cases, including heart rates, diseases, age, sex etc.

SCULLI assumes a linear behavior of the motion between two conjugate PAR images which are a half rotation apart. It seems that this assumption holds for the cases where the rotation speed of the gantry is 0.25 sec/rotation and the heart rate is between 60 and 95 bpm based on our study. More rigorous study is necessary to figure out the limitation of the current approach. A more sophisticated motion estimation method to overcome such limits has been proposed [24]. Performance comparison of the proposed method against other motion correction techniques [1–6] is missing in this paper. Moreover, we were not able to provide reformatted CT images and quantitative measures of coronary arteries from commercially available clinical workstations in this paper.

### Potential applications and future study

CCTA would be the number one application of SCULLI based on its consistent performance in handling motion of coronary arteries. Moreover, there are other applications for which SCULLI could be adopted. First, SCULLI could serve to improve the accuracy of CTP. As pointed out in [15], motion artifacts of myocardial wall could cause degradation in CTP results. Throughout the results of XCAT phantom, Alpha phantom experiments, in-vivo animal and clinical studies, SCULLI showed a consistent performance in handling motion artifacts of myocardial wall. Exploring the impact of SCULLI on CTP would be a very interesting research topic.

Second, we strongly believe, unlike other approaches, SCULLI can be applied to non-contrast scan too and could improve the accuracy of calcium scoring as shown in Fig 14. Again, this is possible due to the advantage of SCULLI being free of segmentation.

Third, SCULLI showed a potential in lung motion correction as presented in Fig 15. We expect that motion artifacts of lung structures become more severe in case of non-gated standard chest scans, in which 0.5 sec or 0.33 sec rotations speeds are typically adopted. Therefore, there will be more rooms where SCULLI comes into play [25–27].

Last but not least, the combinations of noise reduction [28], dual-source system [29], or multi-energy technology and SCULLI would be exciting research topics. We look forward to conducting more clinical studies.

## Conclusions

The result of XCAT phantom experiments demonstrated the overwhelming efficacy of SCULLI by means of visualization of coronary arteries and myocardial wall. Both MSSIM (SCULLI: 0.94, FBP: 0.77, *p*<0.001) and RMSE (SCULLI: 20.27, FBP: 25.33, *p* = 0.01) showed a significant improvement compared to FBP. The result of reader study based on Mocomo phantom experiment also showed a statistically significant improvement by SCULLI (SCULLI: 3.5, FBP: 2.1, *p*<0.001). In addition, SCULLI delivers up to 50% more stable HU values on a profile of myocardial wall throughout the heart cycle. In-vivo animal and clinical study showed that SCULLI performs well and delivers a compelling image quality even at the higher heart rate of 85 bpm. The visualization of coronary arteries, especially most challenging RCA, has been significantly improved. The shadings at myocardial wall are also reduced remarkably. SCULLI showed a great potential in visualization of the whole heart structures, therefore, it can be adopted for routine CCTA and CTP.

## Supporting information

S1 Video 4D FBP image of the aortic valve of case A. The movement of the valve is not clearly visualized at some cardiac phases due to motion artifacts.
(MP4)

S2 Video 4D SCULLI image of the aortic valve of case A. It shows the opening and closing movements of the valve well, which is not the case in FBP image in S1 Video.
(MP4)

S3 Video 4D FBP image of the myocardial wall of case A. Shadings in horizontal direction are prominent.
(MP4)

S4 Video 4D SCULLI image of the myocardial wall of case A. Significantly less horizontal shadings than in FBP (S3 Video) are observed.
(MP4)

## References

[1] Grangeat P, Koenig A, Rodet T, Bonnet S. Theoretical framework for a dynamic cone-beam reconstruction algorithm based on a dynamic particle model. Phys Med Biol. 2002 8;47(15):2611–25. 10.1088/0031-9155/47/15/304 12211208

[2] Van Stevendaal U, von Berg J, Lorenz C, Grass M. A motion-compensated scheme for helical cone-beam reconstruction in cardiac CT angiography. Med Phys. 2008 7;35(7):3239–51. 10.1118/1.2938733 18697549

[3] Isola AA, Grass M, Niessen WJ. Fully automatic nonrigid registration-based local motion estimation for motion-corrected iterative cardiac CT reconstruction. Med Phys. 2010 3;37(3):1093–109. 10.1118/1.3301600 20384245

[4] Bhagalia R, Pack JD, Miller JV, Iatrou M. Nonrigid registration‐based coronary artery motion correction for cardiac computed tomography. Medical Physics. 2012 7;39(7): 4245–4254. 10.1118/1.4725712 22830758

[5] Hahn J, Bruder H, Allmendinger T, Stierstorfer K, Flohr T, Kachelriess M. Reduction of motion artifacts in cardiac CT based on partial angle reconstructions from short scan data. Proc. SPIE 9783; Medical Imaging 2016 Mar: Physics of Medical Imaging 97831A.

[6] Boutchko R, Balakrishnan K, Reutter BW, Gullberg GT. Patient motion correction in computed tomography by reconstruction on a moving grid. 2007 IEEE Nuclear Science Symposium Conference Record; 2007 Oct 26 –Nov 3; Honolulu, United States.

[7] Kim S, Chang Y, Ra JB. Cardiac motion correction based on partial angle reconstructed images in x-ray CT. Medical Physics. 2015 5;42(5): 2560–2571. 10.1118/1.4918580 25979048

[8] Ra JB, Kim S, Lee KY, Rifu T, Yi J, Ahn I, et al. Tomography apparatus and method for reconstructing tomography image thereof. United States patent US9959631. 2018 May 1.

[9] Lee D, Choi J, Lee KY, Shin J, Kim D. Practical simulation with realistic coronary artery motion for validation of motion compensated reconstruction algorithm in CT. ECR 2017: European Congress of Radiology; 2017 Mar 1–5; Vienna, Austria.

[10] Jung JY, Sun M, Yoo SW, Lee G, Lee D, Lee KY, et al. Estimation of effective temporal resolution from high heart rate ECG gated images with a novel temporal enhanced volume reconstruction techniques. SCCT 2015: Society of Cardiovascular Computed Tomography; 2015 Jul 16–19; Las Vegas, United States.

[11] Dutta S, Jung JY, Sun M, Yoo SW, Lee G, Lee D, et al. Improvement in stenosis measurement accuracy in presence of cardiac motion using a novel temporal enhanced volume reconstruction technique. SCCT 2015: Society of Cardiovascular Computed Tomography; 2015 Jul 16–19; Las Vegas, United States.

[12] Jung JY, Sun M, Yoo SW, Yi J, Choi J, Lee D, et al. Assessment of calcium quantification in presence of motion with a novel temporal enhanced reconstruction technique. ECR 2016: European Congress of Radiology; 2016 Mar 2–6; Vienna, Austria.

[13] Park J, Choi J, Lee D, Cho M, Lee KY. Performance of a novel cardiac motion correction in CT scans of 0.33 seconds rotation. CT meeting 2020: 6^th^ international conference on image formation in x-ray computed tomography; 2020 Aug 3–7; Regensburg, Germany. Forthcoming.

[14] Rueckert D, Sonoda LI, Hayes C, Hill DL, Leach MO, Hawkes DJ. Nonrigid registration using free-form deformations: application to breast MR images. IEEE Trans Med Imaging. 1999 8;18(8):712–21. 10.1109/42.796284 10534053

[15] Techasith T, Cury RC. Stress myocardial CT perfusion: An update and future perspective. JACC Cardiovas Imaging. 2011 8;4(8): 905–916. 10.1016/j.jcmg.2011.04.017 21835384

[16] Segars WP, Sturgeon G, Mendonca S, Grimes J, Tsui BM. 4D XCAT phantom for multimodality imaging research. Medical Physics. 2010 9;37(9):4902–15. 10.1118/1.3480985 20964209 PMC2941518

[17] Wang Z, Bovik AC, Sheikh HR, Simoncelli EP. Image quality assessment: from error visibility to structural similarity. IEEE Trans Image Process. 2004 4; 13(4):600–12. 10.1109/tip.2003.819861 15376593

[18] Landis JR, Koch GG. The measurement of observer agreement for categorical data. Biometrics. 1977 3;33(1):159–74. 843571

[19] Lu B, Mao SS, Zhuang N, Bakhsheshi H, Yamamoto H, Takasu J, et al Coronary artery motion during the cardiac cycle and optimal ECG triggering for coronary artery imaging. Invest Radiol. 2001 5;36(5):250–6. 10.1097/00004424-200105000-00002 11323512

[20] Husmann L, Leschka S, Desbiolles L, Schepis T, Gaemperli O, Seifert B, et al Coronary Artery Motion and Cardiac Phases: Dependency on Heart Rate—Implications for CT Image Reconstruction. Radiology. 2007 11;245(2):567–76. 10.1148/radiol.2451061791 17848683

[21] Achenbach S, Ropers D, Holle J, Muschiol G, Werner GD, Werner M. In-Plane Coronary Arterial Motion Velocity: Measurement with Electron-Beam CT. Radiology, 2000 8;216(2):457–63. 10.1148/radiology.216.2.r00au19457 10924570

[22] Mao S, Lu B, Oudiz RJ, Bakhsheshi H, Liu SC, Budoff MJ. Coronary artery motion in electron beam tomography. J Comput Assist Tomogr. 2000 Mar-Apr;24(2):253–8. 10.1097/00004728-200003000-00012 10752887

[23] Hofman MB, Wickline SA, Lorenz CH. Quantification of in-plane motion of the coronary arteries during the cardiac cycle: Implications for acquisition window duration for MR flow quantification. J Magn Reson Imaging. 1998 May-Jun;8(3):568–76. 10.1002/jmri.1880080309 9626870

[24] Kim S, Chang Y, Ra JB. Cardiac image reconstruction via nonlinear motion correction based on partial angle reconstructed images. IEEE Trans Med Imaging. 2017 5;36(5): 1151–61. 10.1109/TMI.2017.2654508 28103549

[25] Kim S, Chang Y, Ra JB. Cardiac motion correction for helical CT scan with an ordinary pitch. IEEE Trans Med Imaging. 2018 7;37(7): 1587–96. 10.1109/TMI.2018.2817594 29969409

[26] Choi J, Lee D, Lee KY, Kim S, Ra JB. Feasibility of cardiac imaging from routine chest CT scans by aid of a novel motion correction algorithm. ECR 2017: European Congress of Radiology; 2017 Mar 1–5; Vienna, Austria.

[27] Choi J, Lee KY, Lee D. Method and apparatus for processing medical image. United States patent US20180103930. 2017 Oct 12.

[28] Seo CW, Rifu T, Lee D, Kim D, Nam S, Lee KY. Tomography apparatus and method of reconstructing cross-sectional image. United States patent US10165989. 2019 Jan 1.

[29] Lee D, Lee KY, Kim D. Tomography apparatus and method of processing tomography image. United States patent US10032295. 2018 Jul 24.

